# The lack of specificity of *sodC*-based PCR for the detection of *Neisseria meningitidis* carriage in pharyngeal swabs from adolescents

**DOI:** 10.1128/spectrum.02779-24

**Published:** 2025-04-08

**Authors:** Dan Duy Pham-Tran, Lex E. X. Leong, Mark McMillan, Andrew Lawrence, Hassen Mohammed, Adam Finn, Helen S. Marshall

**Affiliations:** 1 SA Pathology, Adelaide, South Australia, Australia; 2 Vaccinology and Immunology Research Trials Unit, Women’s and Children’s Health Network, Adelaide, South Australia, Australia; 3 Robinson Research Institute and Adelaide Medical School, The University of Adelaide, Adelaide, South Australia, Australia; 4 Schools of Population Health Science and Cellular and Molecular Medicine, University of Bristol, Bristol, United Kingdom; FIND, Geneva, Switzerland

**Keywords:** carriage, *Neisseria meningitidis*, superoxide dismutase C (*sodC*), carriage

## Abstract

The utility of the superoxide dismutase C (*sodC*) gene for detecting *Neisseria meningitidis* carriage in pharyngeal swab samples is unclear. While it has been explored as an alternative to the capsule transport A gene for detecting invasive strains, its potential as the sole target for real-time PCR in meningococcal carriage screening remains unexplored. This study aimed to assess the utility of using *sodC* as a target for detecting invasive and non-invasive strains of *Neisseria meningitidis* in pharyngeal swabs. Oropharyngeal swab samples collected from adolescents (approximately 15–18 years of age) enrolled in the “B-Part-of-It” cluster-randomized controlled trial (NCT03089086) were selected for this study. Samples were initially screened for the presence of specific *N meningitidis* porin A gene (*porA*) using PCR and then preserved by freezing at −80°C. An assay targeting the *sodC* gene was developed using the locked nucleic acid probe-based method. Upon optimization of the *sodC* assay, 1,092 samples were retested using this assay and compared against the results from the *porA* assays to determine concordance. Out of 1,092 samples tested, 965 (88.4%) were *sodC* positive, with 100% sensitivity and 13% specificity compared to *porA*. The positive predictive values and negative predictive values for the *sod*C assay were 12% and 100%, respectively. When compared to *porA* PCR as the gold standard, the *sodC* assay lacks sufficient specificity to serve as a stand-alone screening assay for the detection of *Neisseria meningitidis* carriage in pharyngeal samples from adolescents.

**IMPORTANCE:**

While the *sodC* assay successfully detects *N. meningitidis*, we identified a limitation in its specificity due to potential cross-reactivity with other organisms, including *Haemophilus* spp., which can result in false positives. This limitation highlights the need for careful interpretation of *Neisseria meningitidis* carriage results, especially in epidemiological studies.

## INTRODUCTION

*Neisseria meningitidis* is a gram-negative diplococcus which can cause life-threatening invasive meningococcal disease (IMD). There are approximately 1.2 million cases and 135,000 global deaths annually (1, 2). The meningococcus primarily resides in the oropharyngeal mucosa of asymptomatic carriers and spreads via aerosol droplets or by direct contact with secretions (1, 2). Carriage rates are typically low in the early years of life, sharply rise in adolescence, and peak around 19 years of age, influenced by the frequency and intimacy of social contacts and exposure to other risk factors in this age group (3). The *Neisseria meningitidis* serogroups most often involved in IMD are A, B, C, X, W, and Y, with B being the most prevalent in high-resource countries (4, 5). Real-time PCR (RT-PCR) is commonly used for detection of both asymptomatic carriage and IMD (5). The most common genes targeted in PCR tests are the capsule transport A (*ctrA*) gene and porin A (*porA*) gene (6). The *ctrA* gene is unique to *Neisseria meningitidis*, and detection of *ctrA* in sterile samples is considered the gold standard for IMD diagnosis (7). However, in the oropharynx, the majority of *Neisseria meningitidis* carried are unencapsulated, commensal strains which lack the capsule biosynthesis genes (8). As such, a PCR assay targeting only the *ctr*A gene is too insensitive for meningococcal carriage study screening (7).

The *porA* gene is considered to be another target for meningococcal carriage screening (9). Despite a highly similar *porA* pseudogene being present in some other *Neisseria* spp., *Neisseria meningitidis porA* specificity can be significantly improved with specific primer and probe optimization (6). RT-PCRs targeting the *porA* gene detect unencapsulated strains of meningococci and have much higher sensitivity than *ctrA*-based assays (6). These properties make *porA* a preferred target for *Neisseria meningitidis* carriage screening. It should be noted, however, that *porA* rarely can be found in *Neisseria gonorrhoeae* and additionally is absent in some meningococci, so there is a small risk of both false-positive and false-negative results (10, 11).

Another target, the superoxide dismutase [Cu-Zn] gene, superoxide dismutase C (*sodC*), has been proposed as an alternative assay target for meningococcal RT-PCR carriage screening (12). The *sodC* gene is described as having high sensitivity and specificity for *Neisseria meningitidis*, and it is present in all known variants including unencapsulated strains (12). These qualities potentially make *sodC* an alternative target for detection of meningococcal carriage. However, other pharyngeal organisms, including *Haemophilus* spp., contain a similar *sodC* gene, which can cause false-positive results (13). A number of previous studies have utilized the *sodC* gene with mixed effectiveness (7, 14). This study aimed to evaluate the performance of a *sodC* RT-PCR to specifically detect *Neisseria meningitidis* in pharyngeal swab samples from adolescents. This study secondarily sought to validate the usability of long-term deep-frozen (−80°C) stored samples from the “B-Part-of-It” meningococcal carriage study (15).

## MATERIALS AND METHODS

### Samples

In the development process, bacterial cultures were utilized for the assay optimization. The specific strains of *Neisseria meningitidis* used were from a mix of American Type Culture Collection (ATCC), National Neisseria Network (Australia), National Collection of Type Cultures (NCTC), and in-house culture controls as follows: Serogroup A ATCC 13077, Serogroup B NNN 09N170, Serogroup C NNN 06N045, Serogroup W135 NNN 10N029, Serogroup Y NNN 07N042, and Serogroup X 48655 ct 16.6. Serogroup X was an SA Pathology in-house control. The *Haemophilus* controls used were *Haemophilus influenzae*, ATCC 49247, ATCC 49766, and NCTC 8468. The *Haemophilus parainfluenzae* control was also from an in-house specimen. For the comparison testing, 1,092 oropharyngeal swab DNA extracts (in extraction plates) from adolescents obtained during the B-Part-of-It (NCT03089086) study conducted in 2017 that had been frozen and stored at −80°C were selected and used. The B-Part-of-It study was a South Australian state-wide cluster-randomized controlled trial in secondary-school students (approximately 15–18 years of age) (*n* = 34,489), conducted in 2017 and 2018, examining the impact of 4CMenB vaccine on the carriage of disease-associated meningococci in adolescents (15).

### *sod*C assay development and validation

DNA was extracted using MagNA Pure 96 Total NA Isolation kit (Roche Diagnostics, Indiana, USA) on a MagNA Pure 96 system (Roche Diagnostics). Primers and probes for the *sod*C assay were based on a previous work (12) with a revised *sod*C probe designed in-house with locked nucleic acids. During an *in silico* assay development, the nucleotide alignments were constructed using the Geneious program, where the primer sequences were aligned against the *sod*C gene of *Neisseria meningitidis* strains with serogroups A, B, C, W, X, and Y and a homologous gene present in multiple *Haemophilus* spp. The primer and probe nucleotide sequences were inputted manually, while sequences for different organisms were obtained as contig from the publicly available international National Center for Biotechnology Information database.

Primer and probe concentration *in vitro* optimization was performed using standard strains of *Neisseria meningitidis* serogroups A, B, C, W, X, Y and *Haemophilus* spp. A laboratory positive control of *Neisseria meningitidis* was utilized to validate the *sod*C assay ability to detect *Neisseria meningitidis* carriage.

*Neisseria meningitidis* laboratory control stains included serogroups A, B, C, W, X, and Y and are maintained by SA Pathology and used for positive controls for other diagnostic tests. Additionally, three *Haemophilus influenzae* strains and one *Haemophilus parainfluenzae* strain isolated from clinical specimens were tested. These 10 controls were tested in duplicate using the *sod*C RT-PCR on a Roche Diagnostic LightCycler 480 Instrument II (Roche Diagnostics).

### Limit of detection study

RT-PCR testing was conducted using the meningococcal B control strain using 10-fold serial dilutions to determine the limit of detection for this assay. The initial working stock was made using a mix of 300 μL lysis buffer, 300 μL PCR-grade water and inoculated with an isolated colony obtained via 1 μL inoculation loop from the chocolate agar culture plate of a serogroup B control strain. Fifty microliters of this mixture was then added to 450 μL total volume of equal parts lysis buffer and PCR-grade buffer to create a mixture with 1:10 concentration of the previous. This process was then repeated to produce serial dilutions from 10^0^ to 10^−5^. Inhibition control primers and probe were added to the optimized master mix for all tests performed.

### B-Part-of-It sample testing

Following assay optimization, nasopharyngeal swab samples (*n* = 1,092) from the B-Part-of-It study were tested. Primers and probes used are listed in Table 1. The PCR procedure included an initial denaturation at 50°C for 15 minutes, followed by denaturation at 95°C for 10 minutes. This was followed by 45 cycles of 95°C for 10 seconds, annealing at 55°C for 15 seconds, and extension at 60°C for 30 seconds. The procedure was consistent with the *porA* assay used in the B-Part-of-It carriage study to ensure comparability of results.

**TABLE 1:** Oligonucleotide sequences for *sodC* primers and probe

Name	Alignment	Source
Nm *sod*C Fwd 351	GCA CAC TTA GGT GAT TTA CCT GCA T	Reference (14)
Nm *sod*C Rev 478	CCA CCC GTG TGG ATC ATA ATA GA	Reference (14)
Nm *sod*C Pb 387	(FAM)-CAT GAT GGC ACA GCA ACA AAT CCT GTT T-(BHQ1)	Reference (14)
Nm *sod*C Pb Mod	(6FAM) TT[+G] CAT GAT GG[+C] ACA GCA ACA AA[+T] CCT GT (BHQ1)	Modification of Nm *sod*C PB 387 to create locked nucleic acid probe

### Statistical analyses

A 2 × 2 contingency table was constructed to compare the *sodC* assay results against the *porA* test, used as the “gold standard” for comparison. Diagnostic metrics, including sensitivity, specificity, positive predictive value (PPV), and negative predictive value (NPV), were calculated. The analysis was performed using Stata 17.0, adhering to standard diagnostic accuracy methods.

## RESULTS

### Validation of sample storage conditions

In order to determine whether there was any significant degradation of DNA in the stored samples from the 2017 study, an extraction plate was retested with the *porA* assay. All samples retested as positive using the *porA* test, with cycle threshold (CT) values all within one cycle from the 2017 testing, confirming that little or no degradation of DNA in the samples was evident. No cross contamination from positive wells was observed. On the extraction plate, a total of eight samples tested positive for *porA*. Additionally, the limit of detection testing showed that at a dilution of 10⁻³, there was insufficient genetic material for PCR using the LightCycler 480 to detect the presence of *Neisseria meningitidis*.

### Limit of detection

The PCR limit of detection was 67 CFU/reaction or 536 CFU/mL.

### Locked nucleic acid probe alignments and control results

**Fig 1:**
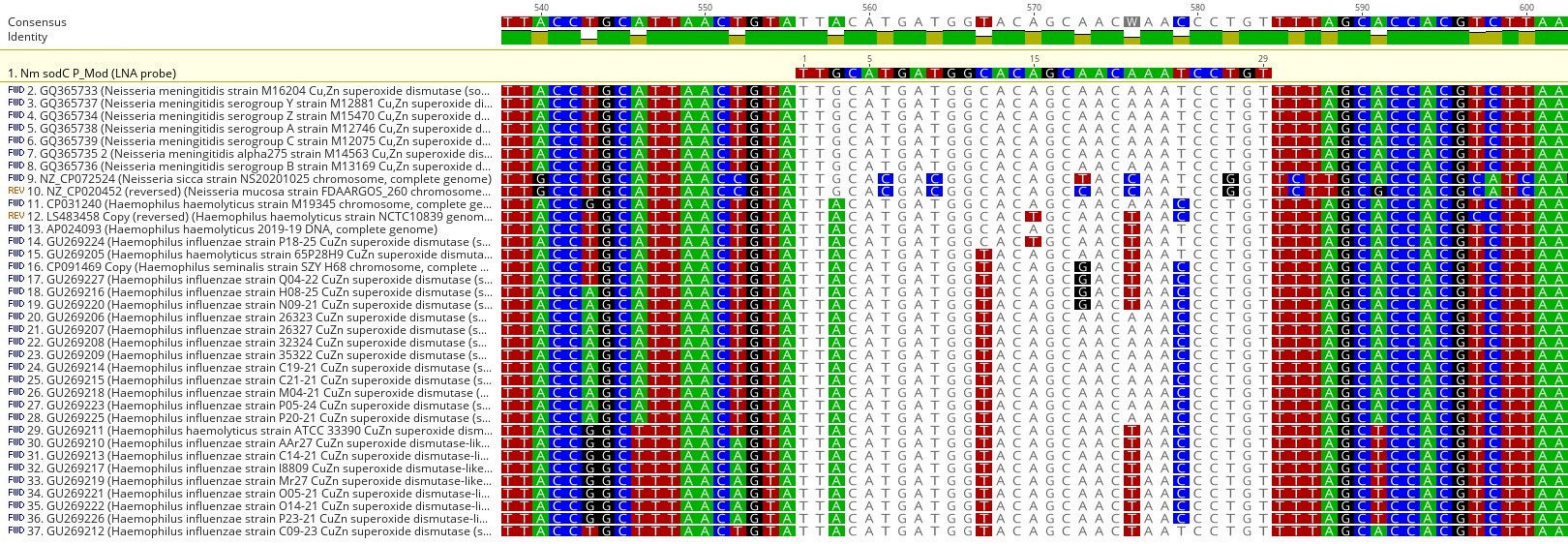
Alignments of the LNA probe. The “locked” nucleotides are the highlighted G, C, and T bases for the *Neisseria meningitidis* sequences. Note: other organisms do not contain the combination of all three.

From the sequence alignments on Geneious, there are two bases within the probe’s binding area and a G base just outside of it. The binding area was shifted to the left relative to the sequence to include the G base, and then these three specific nucleotide acids were “locked.” As seen with the locked nucleic acid (LNA) probe’s alignment, only meningococcal strains contained all three bases with no non-Nm bacterial strains listed containing the full alignment. This probe alignment was also entered into a Basic Local Alignment Search Tool (BLAST) search to determine the most homologous non-meningococcal organisms and the least homologous meningococcal strains. These results showed that there were no complete matches for the LNA probe against other bacteria except *Neisseria meningitidis*, where all recorded strains aligned with the target sequence perfectly (Fig. 1). Testing the same set of control strains using the LNA probe showed that there was very good amplification for all the *Neisseria meningitidis* strains except for the serogroup W strain, which remained negative. The previously positive *Haemophilus* spp. had higher crossing points but were still regarded as being detected.

### Sample testing

One thousand and ninety-two DNA extracts were retrieved from the DNA extraction plates stored at −80°C. When compared with the data obtained from the B-Part-of-It study, all 116 previously *porA*-positive samples were also *sodC* positive. Additionally, 849 samples that were previously *porA* negative in the original 2017 study tested *sodC* positive. Using *porA* RT-PCR as the standard for *Neisseria meningitidis* carriage detection, the *sodC* assay demonstrated a sensitivity of 100% but only a specificity of 13%. The PPVs and NPVs were calculated, with the assay having a PPV of 12% and an NPV of 100%. This means that only 12% of the *sodC*-positive samples were true positives (using *porA* as the gold standard) for carriage of meningococci, while all *sodC* negatives were true negatives (Table 2).

**TABLE 2:** Comparison of *sodC* test results with the original 2017 *porA* test results (*n* = 1,092)

Criteria	Original *porA* positive	Original *porA* negative	Total
*sodC* positive	116	849	965
*sodC* negative	0	127	127
Total	116	976	1,092

**Fig 2:**
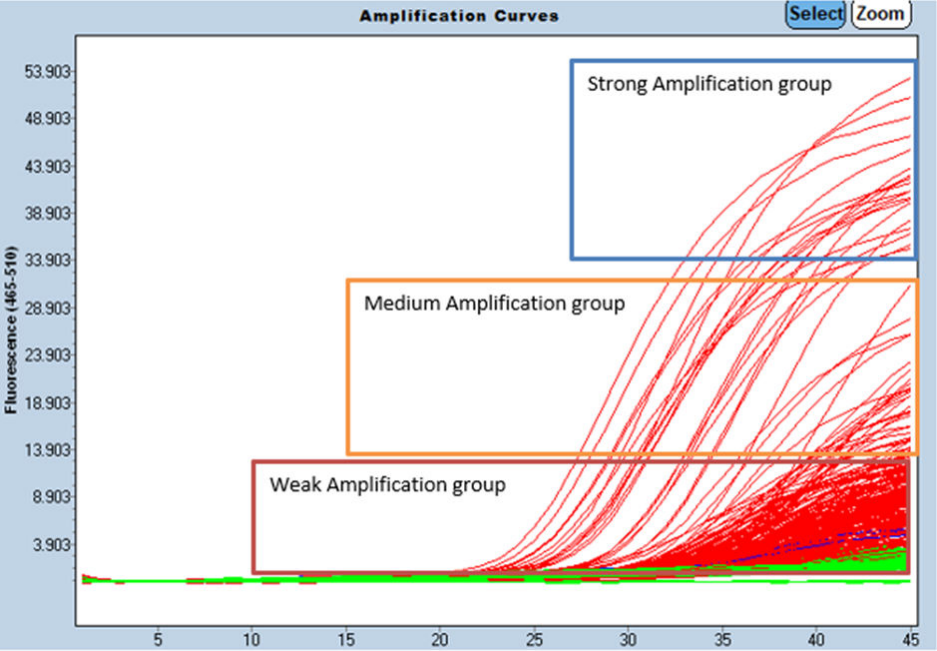
*SodC* amplification curves of three plates that were tested at the same time, with three groupings, shown as fluorescence over cycles.

The positive *sodC* RT-PCR results can be categorized into three groups based on strength of amplification (Fig. 2). There was a group characterized by strong fluorescence with good amplification curves which were all *sod*C and *por*A positive. The other two groups included the “medium” amplification group and a weaker, flatter amplification group, the latter consisting of a high proportion of *sod*C-positive/*por*A-negative samples.

## DISCUSSION

This study confirmed the *sodC* assay will detect *Neisseria meningitidis* but with unreliable specificity when compared to *porA* PCR as the standard. This means *sod*C positives cannot be confidently attributed to the presence of *Neisseria meningitidis* alone making accurate identification with only *sodC* RT-PCR impossible. This study suggested it was only possible to detect a true positive when results from *porA* RT-PCRs were also available if *porA* is considered as the standard. Consequently, using the *sodC* assay, as described in this study for only meningococcal carriage screening, is ineffective due to non-specificity. However, if utilized within a multiplex assay where other bacterial species such as *Haemophilus influenzae* are already targeted, the non-specificity of the assay can be mitigated while also improving the overall accuracy. Additionally, *sodC* is an effective diagnostic target for IMD, detecting both encapsulated and non-encapsulated *Neisseria meningitidis*, unlike *ctrA*, which may fail to identify non-groupable strains.

Given the constraints of the study design, we were unable to test for the presence of *H. influenzae* and other *sodC* pseudogene carriers, as participant consent was restricted to screening for meningococcal carriage. Despite these limitations, this study demonstrated that the *sodC* assay as described can detect *Neisseria meningitidis* carriage from pharyngeal swab sample DNA extracts from adolescents. However, although the *sodC* assay could detect meningococcal carriage with the same sensitivity as *porA*, it exhibited significantly lower specificity, with 88% of *sodC* positives being considered false positives when using *porA* as the standard.

The two available serogroup W samples from the original B-Part-of-It study used in this study were found to be *sodC* positive. However, during validation, the W control strain failed to amplify with the *sod*C assay, despite testing positive with both the *porA* and *ctrA* assays. Sequence alignments and BLAST searches confirmed that all known meningococcal strains, including W, contain the *sod*C gene and should amplify when tested. The reason for the W control strain’s failure to amplify remains unclear. Separate from this study, further investigation, including whole-genome sequencing, is needed to explore potential *sod*C deficiency in meningococcal W strains, as this was beyond the scope of the current study.

To check for DNA degradation from storage at −80°C from the 2017 study, 91 samples were retested for *por*A. The results replicated the positives recorded in the B-Part-of-It data from 2017. This confirmation rules out DNA extract degradation and cross contamination as factors attributing to the differences between *sod*C and *por*A assays. These findings also confirm the usability of DNA extracts stored frozen for up to 5 years for RT-PCR-based research. However, the impact of longer-term storage remains unknown. While this observation is based on testing only 91 samples with the *por*A assay, uniform handling and storage procedures for all plates suggest these issues are unlikely to be significant across the entire sample set tested.

When analyzing the fluorescence curves of the sample testing, a subset consisting of *sod*C-positive/*por*A-positive results showed significantly higher amplification, but this was observed in only a portion of the confirmed *sod*C-positive samples with both the “mid-strength” and “weak” amplification groups containing mixtures of *sod*C-positive but *por*A-positive and *por*A-negative samples. The weaker and flatter amplification curve samples may have been due to cross-reactivity to other organisms like *Haemophilus* spp., which share homologous sequences in the probe binding and or primer sites. Without further sequencing analysis to confirm this, it is difficult to draw firm conclusions.

In contrast to previous studies (7, 14), no specific cycle threshold limit was set for the *sodC* assay, as it was directly compared to the protocol used in the B-Part-of-It study. In this study, any *por*A amplification observed with a CT value up to and including 40 was considered positive. For the original B-Part-of-It study, *por*A RT-PCR, positive *por*A samples which also yielded a culture of *Neisseria meningitidis* (therefore no question about being positive) showed a normal distribution of CT values, except for a significant number having a CT value of 40 (unpublished data). This suggests that pharyngeal samples likely will contain a wide range of quantities of specific meningococcal DNA due to differences in degrees of colonization (16, 17). A large proportion of the samples tested (88.4%) yielded a positive *sod*C result, but only 12% were also positive for *por*A. While all *por*A-negative and *sod*C-positive results were considered false positives in this study, it is important to note that *por*A-deficient meningococcal isolates, both encapsulated and non-encapsulated, are rarely reported (10). Therefore, it is possible that the *sod*C assay detected *por*A-deficient meningococci among these samples. However, this possibility alone does not account for the large discrepancy between the *por*A-negative and *sod*C-positive results.

Despite these issues as discussed, *sodC* as a target is proven to detect *Neisseria meningitidis* in adolescents, with the assay developed having potential for improvement and possible niche usage in the current form. While the LNA probe showed no noticeable improvement on assay specificity, a reverse-complement LNA probe could have an effect and, despite the lack of specificity, the current assay can be used in a multiplex assay where this weakness is mitigated.

### Conclusion

The *sod*C assay was demonstrated to detect *Neisseria meningitidis*; however, it lacks specificity for identifying meningococci in the context of pharyngeal carriage. This limitation arises because the assay possibly also detects other organisms present in pharyngeal swab samples. This cross-reactivity with other organisms, including *Haemophilus* spp. can lead to false-positive results, reducing the reliability of the assay for the identification of *Neisseria meningitidis* carriage. Therefore, while the *sod*C assay could be used as a tool in the detection of *Neisseria meningitidis* carriage, its lack of specificity necessitates careful interpretation of carriage results, especially in the context of epidemiological studies.
